# The prognostic implication of visual acuity at the time of uveal melanoma diagnosis

**DOI:** 10.1038/s41433-022-02316-8

**Published:** 2022-11-24

**Authors:** Elin Asplund, Maria Fili, Tony Pansell, Rune Brautaset, Maria Nilsson, Gustav Stålhammar

**Affiliations:** 1 Department of Clinical Neuroscience, Division of Ophthalmology and Vision, Unit of Optometry, Karolinska Institutet, Stockholm, Sweden; 2 Department of Clinical Neuroscience, Division of Ophthalmology and Vision, Unit of Ocular Oncology and Pathology, Karolinska Institutet, Stockholm, Sweden; 3 St. Erik Eye Hospital, Stockholm, Sweden; 4 Department of Clinical Neuroscience, Division of Ophthalmology and Vision, Marianne Bernadotte Centre, Karolinska Institutet, Stockholm, Sweden

**Keywords:** Uveal diseases, Outcomes research

## Abstract

**Background:**

Visual outcomes after primary tumour treatment of uveal melanoma (UM) have been investigated repeatedly. This study evaluates the correlation between best-corrected visual acuity (BCVA) before treatment with clinicopathological factors and patient survival.

**Subjects/Methods:**

Pre-treatment BCVA was examined in relation to tumour dimensions and location, and survival in a retrospective cohort of 1809 patients who underwent plaque brachytherapy. BCVA was also correlated to tumour histological factors in a second cohort of 137 enucleated eyes.

**Results:**

The mean BCVA of the tumour eye prior to plaque brachytherapy was LogMAR 0.42 (SD 0.46). Patients with low BCVA (LogMAR ≥ 1.00) did not differ in age (*p* = 0.19) and had similar frequency of ciliary body involvement (*p* = 0.99) but had tumours with greater apical thickness (*p* < 0.0001), greater diameter (*p* < 0.0001) and shorter distance to the optic disc and fovea (*p* < 0.0001). There were no significant relations between low BCVA and any of 13 examined tumour histological factors at a Bonferroni-corrected significance level (*p* > 0.004). Patients with low BCVA had greater incidence of UM-related mortality in competing risk analysis (*p* = 0.0019) and shorter overall survival (*p* < 0.0001). Low BCVA was also associated with increased hazard ratio (HR) for UM-related mortality in univariate analysis (HR 1.5, 95% confidence interval 1.2 to 1.9), but not in multivariate analysis with tumour size and location as covariates.

**Conclusions:**

UM patients with low BCVA before primary tumour treatment have a worse prognosis, likely related to increased tumour dimensions. Future studies should examine the prognostic significance of BCVA in relation to macula-involving retinal detachment and genetic factors.

## Introduction

Uveal melanoma (UM) is the most common primary intraocular malignancy, affecting more than 7000 individuals each year worldwide [1]. UMs most commonly arise in the choroid (90% of cases), followed by the ciliary body (6%) and iris (4%) [2]. Within 15 years from diagnosis, 32 to 45% of patients succumb to metastatic disease, with no survival difference after enucleation or eye-preserving plaque brachytherapy of eyes with medium-sized tumors [3–6].

Many studies have reported short and long-term visual outcomes after plaque brachytherapy and other eye-preserving treatment modalities [7–16]. These indicate that one-half of patients lose six or more lines of visual acuity (VA) three years after treatment [15]. At 10 years, 50 to 60% patients have Snellen <20/200 and 75% have abnormal visual field (VF) sensitivity (10-2 testing) [7, 9, 12, 17]. Further, greater tumour thickness, shorter distance to the fovea, subretinal fluid, worse baseline VA, non-Caucasian ethnicity, specific tumour shapes and a simultaneous diabetes diagnosis have been associated with increased risk for poor VA, with more than 99% of patients suffering from Snellen <20/200 if all factors are present [18].

Histologically, UMs are identified as dome-shaped, mushroom-shaped or lobulated masses of atypical melanocytes with varying degrees of pigmentation and vascularity [19–24]. Typically, eyes that have been enucleated with UM are examined by dedicated ophthalmic pathologists or by pathologists with ophthalmic pathology as a special interest. The largest basal diameter and apical thickness of a UM together with information about the presence or absence of ciliary body involvement and extraocular tumour extension form the basis for the American Joint Committee on Cancer (AJCC) T-categories [25]. For staging, information about the presence or absence of detectable distant metastases is required. Multiple studies have described the correlations between these factors and patient survival, as well as between a range of clinical and histological factors including but not limited to tumour size, cell type, growth patterns, presence of fluid-conducting extracellular matrix patterns known as vasculogenic mimicry, optic nerve head infiltration and tumour necrosis [19, 22, 26–40]. Factors such as subretinal fluid, tumour penetration of Bruch’s membrane and tumour retinal invasion have been shown to correlate with loss of ≥5 lines of Snellen visual acuity at last follow-up after plaque brachytherapy [9]. Further, reduced visual acuity prior to enucleation of an eye with UM has been found to correlate with postlaminar optic nerve invasion, which in turn is a marker for poor prognosis [29].

One could perhaps assume that a correlation between low VA and large tumour size, and thereby poor prognosis, is self-evident and that such a basic correlation as the prognostic importance of visual acuity in the tumour eye would have been examined repeatedly. A reduced VA in UM could however be caused by several factors not related to tumour size or location, including but not limited to retinal detachment, tumour infiltration of the retina, bleeding, necrosis, inflammation, and cataract from direct contact between tumour and lens. And quite surprisingly, we are only aware of one previous study that has examined the implication of VA before treatment for patient survival, and none that has included histological characteristics. In a cohort of UM patients treated with plaque brachytherapy published in 2018, Snellen visual acuity ≤ 20/200 (LogMar ≥ 1.00) was associated with increased hazard ratio (HR) for metastasis in univariate Cox regression [41]. VA was however not entered into multivariate analyses and no point estimates and no survival curves based on neither competing risk analysis nor actuarial methods were provided. Herein, we therefore compare these factors in a large, combined cohort of patients treated with plaque brachytherapy or enucleation at a Swedish ocular oncology service with national coverage.

## Methods

### Two cohorts with a total of 1946 patients were included in this study

The first cohort consisted of clinical data from consecutive patients with UM treated with plaque brachytherapy between the years 1980 and 2017 at St. Erik Eye Hospital, Stockholm, Sweden. Pre-established inclusion criteria were: Availability of all the clinical data parameters detailed below. Exclusion criterium was: Less than three years of follow-up. The apical thickness of all tumours had been measured with A and B-scan ultrasonography at diagnosis. Tumour diameters and distances to the optic disc and fovea had been measured upon examination with slit-lamp biomicroscopy and wide-field fundus photographs at the same occasion. Plaque brachytherapy was typically initiated one to three weeks later.

The second cohort consisted of paraffin-embedded and formalin-fixed eyes that underwent primary enucleation for UM between the years 1985 and 2010. For diagnostic purposes, these had previously been stained with haematoxylin and eosin, as well as periodic acid-Schiff (PAS) without haematoxylin counterstain. Inclusion criteria were: Histologically proven melanoma originating from the choroid or ciliary body, and availability of all the clinical data parameters detailed below. Exclusion criteria were: No or too little tumour tissue represented in section, extensive tumour necrosis, haemorrhage or inflammation, heavily pigmented tumour affecting visual examination and suboptimal staining results, determined by positive and negative internal and external controls, and patient still alive (which was a term of our ethical approval). One representative section from each eye was digitally scanned at × 400 using Nano Zoomer 2.0 HT scanner (Hamamatsu Photonics K.K., Hamamatsu, Japan) as described previously [42–44]. The digitalized slides were examined by two authors (EA and GS). In each section, the following thirteen parameters were evaluated: (1) Tumour cell type, (2) degree of tumour pigmentation (heavy tumour pigmentation defined as cell borders, cytoplasmic organelles and nuclei obscured by pigment in >50% of tumour), (3) presence and (4) diameter of necrotic areas within the tumour, (5) presence of vasculogenic mimicry, (6) presence of subretinal fluid, (7) presence of extrascleral tumour growth (extraocular extension), (8) diameter of extraocular growth, (9) tumour penetration of Bruch’s membrane, and (10) tumour infiltration of the optic nerve head, (11) ciliary body, (12) iris and/or (13) anterior chamber angle.

For both cohorts, clinical data on patient sex, age at diagnosis, best corrected visual acuity (BCVA), tumour thickness, tumour diameter, tumour distance to the optic disc and fovea, presence of retinal detachment (as observed upon slit-lamp biomicroscopy, macular involvement not specified in data), other ocular diseases (e.g., cataract, age-related macular degeneration, diabetic retinopathy and glaucoma), AJCC T category, date of diagnosis and vital status at last follow-up (alive, dead from UM or dead from other cause) were obtained from our treatment registry [25]. The registry is regularly updated with dates and causes of death from the national Cause of Death Registry. Previously, it has been estimated to capture more than 95% of UM patients in the country [45]. To reduce the number of classification errors, e.g., death from metastatic UM coded as death from metastatic cutaneous melanoma, and to validate the clinical data, the registry is crosschecked against other diagnoses in the national Cancer Registry, against results from metastatic screening with ultrasound or computed tomography (CT) of the abdomen/liver every 6 months for the first 5 years after diagnosis, and against hospital medical records, as described previously [4, 46, 47]. As the exact date of death could only be approximated within a few weeks or months based on medical records for some of the very oldest entries in the registry, overall survival curves in relation to BCVA were generated by the actuarial life table method in one-year intervals, as described below.

The study follows the tenets of the Declaration of Helsinki and the research group’s internal data security policy for sensitive data. Ethical permission was obtained from the Regional Ethics Review Board in Stockholm (reference 2019–03485) and from the Swedish Ethical Review Authority (record number 2020-02835). According to the approved ethics application, the requirement for written informed consent was waived for the first cohort because this was a retrospective study that did not require collection of identifiable health information including patient names, personal identifiers, addresses, other contact details or photographs, and did not affect treatment or follow-up of the patients. Similarly, the requirement for written informed consent was waived for the second cohort as only deceased patients were included.

### Visual acuity

Best corrected visual acuity (BCVA): Each patient’s VA had been measured according to a standardized methodology one to three weeks prior to treatment by an optometrist or ophthalmic nurse at the Ocular Oncology Service, St. Erik Eye Hospital. A KM-chart in an illuminated light box was used and patients tested at a distance of 3 m [48]. The BCVA recorded was the smallest line at which five of five or six of seven letters were correctly identified after subjective refraction and corrected in a trial frame. All VA measurements reported refer to the tumour eye only (monocular) and were initially recorded on the decimal scale after which they were LogMAR-converted. Patients could wear their own spectacles if appropriate. The method for BCVA measurement was identical throughout the studied period.

Low and high BCVA: The measured BCVAs were dichotomized so that high BCVA was defined as LogMAR <1.00 (>0.1 on the decimal scale); and low BCVA as LogMAR ≥ 1.00 (≤0.1 on the decimal scale), following a previously used classification [9, 49]. In analysis of overall survival and the cumulative incidence of UM-related mortality, we also divided patients into three categories based on BCVA (high LogMAR ≤0.301 (≥0.5 on the decimal scale); intermediate LogMAR >0.301 to <1.00 (<0.5 to >0.1 on the decimal scale): and low LogMAR ≥1.00 (≤0.1 on the decimal scale).

### Vasculogenic mimicry

In the second cohort, patterns of microvascular loops and networks were assessed in a light microscope through a green narrow band pass filter according to the method described by Folberg et al. [31]. For statistical purposes, tumours were categorized into two groups based on the presence or absence of patterns with the strongest prognostic association: Extracellular networks, closed loops, arcs with branching, or any combination of these [31, 32, 50]. This definition replicates one of our previous publications, in which these patterns correlated strongly to digitally measured density of Periodic acid-Schiff structures, loss of BAP-1 expression, gene expression class 2 and short metastasis-free survival [51]. Further, the prognostic significance of the presence of loops, networks and combined patterns have been verified in several publications from other laboratories [36, 52, 53].

### Statistical methods

Differences with a *p* < 0.05 were considered significant, all p-values being two-sided. Shapiro-Wilk tests were used to evaluate the deviation of continuous variables from normal distribution. If the test was significant (*p* < 0.05), the Mann Whitney U tests were used, otherwise Student’s *t*-test were used. Violin plots and linear regression were used to evaluate tumour thickness, diameter and distance to the optic disc and fovea in relation to BCVA. Uni- and multivariate Cox regression HRs for UM-related mortality in relation to BCVA, tumour dimensions and distance to the optic disc and fovea were calculated. Cumulative incidence function estimates from competing risks data were plotted with the cmprsk package for R, and the equality of survival distributions was tested with Gray’s test for equality [54]. Overall survival curves in relation to BCVA were generated by the actuarial life table method in one-year intervals, and the Wilcoxon (Gehan) test was applied. In analysis of low BCVA in relation to the 13 examined histological parameters, we used contingency tables and Pearson chi-square (χ^2^) tests (if all fields had a sample of >5) or Fisher’s exact tests (if any field had a sample of <5). As this involved a large number of tests without preplanned hypotheses which would have increase the risk for type I error, Bonferroni correction was applied and the significance level was reduced to 0.004 (0.05 divided by 13) [55]. BCVA levels less than 0.10, 20/200 or 1.00 (decimal scale, Snellen and LogMAR, respectively) were translated into numerical values according to standards from the Swedish National Quality Registry for Cataracts, in which counting fingers at a distance of 4 m is recorded as 0.08 on the decimal scale, hand movements as 0.04, perception and localization of light as 0.01 and amaurosis as 0.001. All statistical analyses except competing risk survival analyses were performed using IBM SPSS statistics version 27 (Armonk, NY) and GraphPad Prism version 9.3.0 (San Diego, CA, USA).

## Results

### First cohort

One thousand eight hundred and seventy patients met the inclusion criteria, of which 61 were excluded for lack of follow-up data. One thousand eight hundred and nine patients remained in the study. Of the 1809 patients, 896 (50%) were men and 913 (50%) were women. The mean age at diagnosis was 63 years (SD 14). The mean tumour thickness was 5.7 mm (SD 2.9) and the mean diameter 10.9 mm (SD 3.9 mm). The mean BCVA of the tumour eye prior to treatment was 0.42 (SD 0.46). One thousand five hundred and eight patients (83%) had high BCVA (LogMAR < 1.00) whereas 301 patients (17%) had low BCVA (LogMAR ≥ 1.00).

Eight hundred and forty-five patients (47%) had deceased before the end of follow-up. Median follow-up for the 964 survivors was 10.1 years (SD 8.0, Table 1).

**Table 1:** Clinical characteristics of patients and tumours in the first cohort.

***n***	1809
**Mean age at diagnosis, years (SD)**	63 (14)
**Sex,** ***n*** **(%)**	
Female	913 (50)
Male	896 (50)
**Mean tumour thickness, mm (SD)**	5.7 (2.9)
**Mean tumour diameter, mm (SD)**	10.9 (3.9)
**BCVA, mean LogMAR (SD)**	0.42 (0.46)
**BCVA, classification**	
High (LogMAR < 1.00), *n* (%)	1508 (83%)
Low (LogMAR ≥ 1.00), *n* (%)	301 (17%)
**Mean tumour distance to the optic disc, mm (SD)**	3.9 (3.3)
**Mean tumour distance to the fovea, mm (SD)**	3.5 (3.5)
**Median follow-up, years (SD)**	10.1 (8.0)

SD, standard deviation. BCVA, best-corrected visual acuity of tumour eye.

**Fig. 1:**
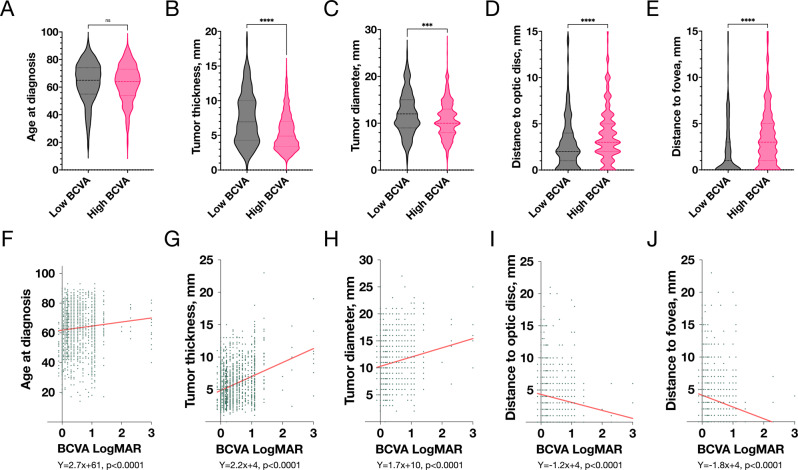
Correlation between visual acuity and clinical factors. **A** The median patient age at diagnosis was similar between patients with high and low best corrected visual acuity (BCVA). Patients with low BCVA had **B** tumours with greater apical thickness, **C** with greater diameter, **D** with shorter distance to the optic disc, and **E** with shorter distance to the fovea. In linear regression, BCVA as a continuous variable correlated with all of **F** patient age at diagnosis, **G** tumour apical thickness, **H** tumour diameter, **I** with shorter distance to the optic disc, and **J** fovea. ***significant on the <0.001 level. ****significant on the <0.0001 level. NS, non-significant.

Patients with high and low BCVA were of similar age, had similar frequency of retinal detachment (any portion of the retina) and ciliary body involvement observable in slit-lamp biomicroscopy and/or ultrasonography. All other clinical factors (tumour thickness, diameter, distance to optic disc, and distance to fovea) differed significantly. In linear regression, decreasing BCVA also correlated with older patient age (Fig. 1).

### Survival analyses

In univariate Cox regression, lower BCVA, and greater tumour apical thickness, tumour diameter and tumour distance to the optic disc were associated with increased HRs for UM-related mortality. In multivariate regression, only tumour thickness and diameter retained their significance (Table 2).

**Table 2:** Cox regression, hazard for melanoma-related mortality.

	B	S.E.	Wald	*p*	Exp(B)	95 % CI lower	95 % CI upper
**Univariate**							
LogMAR BCVA, per increment	0.4	0.1	20.5	**<0.0001**	1.5	1.2	1.7
LogMAR BCVA, low versus high	0.4	0.1	11.8	**0.001**	1.5	1.2	1.9
Tumour thickness, per mm	0.2	0.02	114.8	**<0.0001**	1.2	1.1	1.2
Tumour diameter, per mm	0.2	0.01	204.8	**<0.0001**	1.2	1.2	1.2
Tumour distance to optic disc, per mm	0.03	0.02	4.7	**0.03**	1.0	1.0	1.1
Tumour distance to fovea, per mm	0.03	0.02	3.5	0.06	1.0	1.0	1.1
**Multivariate**							
LogMAR BCVA, per increment	0.06	0.1	0.2	0.67	1.1	0.8	1.4
Tumour thickness, per mm	0.1	0.03	6.5	**0.01**	1.1	1.0	1.1
Tumour diameter, per mm	0.1	0.02	43.5	**<0.0001**	1.1	1.1	1.2
Tumour distance to optic disc, per mm	0.04	0.03	1.4	0.24	1.1	1.1	1.2
Tumour distance to fovea, per mm	−0.02	0.03	0.5	0.48	1.0	0.9	1.0

BCVA, best corrected visual acuity of tumour eye. AJCC, American Joint Committee on Cancer. Boldface indicates significant associations.

**Fig. 2:**
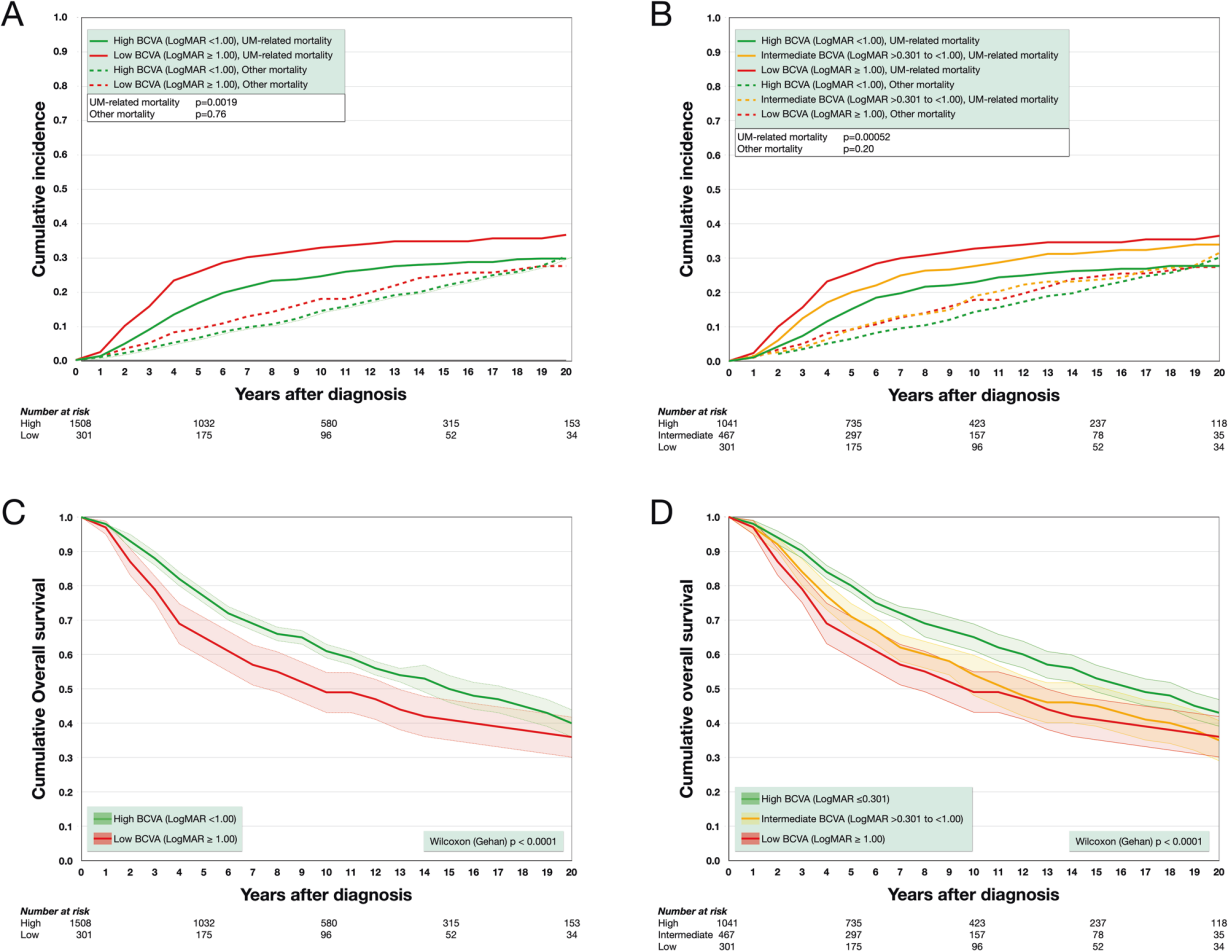
Cumulative incidence of UM related mortality and overall survival. **A** Patients with low BCVA (LogMAR ≥ 1.00, ≤0.1 on the decimal scale) had significantly greater incidence of UM-related mortality than patients with high BCVA (LogMAR < 1.00, >0.1 on the decimal scale, Gray’s test for equality *p* = 0.0019). **B** Similarly, if dividing patients into three categories based on BCVA, patients had greater incidence of UM-related mortality with decreasing BCVA category (high LogMAR ≤0.301, ≥0.5 on the decimal scale; intermediate LogMAR >0.301 to <1.00, <0.5 to >0.1 on the decimal scale; low LogMAR ≥ 1.00, ≤0.1 on the decimal scale, *p* = 0.00052). **C** In actuarial life table analysis, patients with low BCVA had significantly shorter overall survival than patients with high BCVA (Wilcoxon (Gehan) *p* < 0.0001). **D** Similarly, if dividing patients into three categories based on BCVA, patients had shorter overall with decreasing BCVA category (*p* < 0.0001). Coloured areas represent 95% confidence intervals.

In cumulative incidence function estimates of UM-related mortality from competing risks data, patients with low BCVA had significantly greater incidence of UM-related mortality than patients with high BCVA (Gray’s test for equality *p* = 0.0019, Fig. 2A). Similarly, when dividing patients into three categories based on BCVA, patients had greater incidence of UM-related mortality with decreasing BCVA category (*p* = 0.00052, Fig. 2B).

Patients with low BCVA also had significantly shorter overall survival than patients with high BCVA (Wilcoxon (Gehan) *p* < 0.0001, Fig. 2C). When dividing patients into three categories based on BCVA, patients had shorter overall survival with decreasing BCVA category (*p* < 0.0001, Fig. 2D).

### Second cohort

One hundred and fifty-one eyes met the inclusion criteria, of which fourteen were excluded (*n* = 7 patient still alive, *n* = 6 no or too little tumour tissue represented in section, and *n* = 1 the represented tumour was completely necrotic). One hundred and thirty-seven eyes remained in the study. Of these 137 patients, 77 (56%) were men and 60 (44%) were women. The mean age at diagnosis was 65 years (SD 13). The mean tumour thickness was 8.0 mm (SD 3.3) and the mean diameter 14.0 mm (SD 4.6 mm). The mean BCVA of the tumour eye prior to enucleation was 0.75 (SD 0.68). Eighty-four patients (61%) had high BCVA (LogMAR < 1.00) whereas 53 patients (39%) had low BCVA (LogMAR ≥ 1.00).

At the time of UM diagnosis, thirteen patients (9%) had other ocular diseases that may have contributed to a decreased BCVA. Of these, six (4%) had cataract, five (3%) had glaucoma, one (1%) had retinal scarring from a tuberculosis infection in childhood and two (1%) had mild diabetic retinopathy (Supplemental Table). An additional eight patients (6%) had undergone previous cataract surgery in the tumour eye. Three patients (2%) had suffered from recurring migraine episodes and visual aura for several decades. All patients had deceased before the end of follow-up. Ninety-one patients had died from metastatic UM. Median follow-up for the 46 patients that had died from other causes was 10.0 years (SD 5.4).

### Histological findings

Upon histological examination, 39 tumours (28%) were composed of >90% epithelioid cells, 71 (52%) of mixed cell types and 27 (20%) of >90% spindle cells. Fourteen tumours (10%) had necrotic areas. Vasculogenic mimicry was identified in 48 tumours (35%). In contingency tables, there were no statistically significant relationships between low BCVA and any one of the examined tumour histological factors at the Bonferroni-corrected significance level (χ^2^ or Fisher’s exact p > 0.004, Supplemental Fig., Table 3).

**Table 3:** Distribution of tumour histological factors over all patients, and patients with low and high BCVA.

	All patients, *n*	High BCVA (LogMAR < 1.00), *n*	Low BCVA (LogMAR ≥ 1.00), *n*	*p*
Tumour cell type				0.88
Epithelioid	39 (28)	24	15	
Mixed	71 (52)	45	26	
Spindle	27 (20)	15	12	
Heavy tumour pigmentation	14 (10)	9	5	0.81
Tumour necrosis	14 (10)	9	5	0.81
Vasculogenic mimicry present	48 (35)	35	13	0.048
Subretinal fluid	77 (56)	42	35	0.089
Extraocular extension	5 (4)	2	3	0.37
Bruch’s membrane tumour penetration	21 (15)	11	10	0.35
Optic nerve head infiltrated by tumour	10 (7)	6	4	1.0
Ciliary body infiltrated by tumour	25 (18)	15	10	0.85
Iris infiltrated by tumour	8 (6)	5	3	1.0
Anterior chamber angle infiltrated by tumour	10 (7)	6	4	1.0

BCVA, best corrected visual acuity of tumour eye.

## Discussion

In this paper, we have examined correlations between pre-treatment visual acuity, clinical characteristics, patient survival and histopathological findings in eyes with UM. Patients with low BCVA had tumours with significantly greater apical thickness, diameter and significantly shorter distance to the optic disc and fovea. Patients with low BCVA also had significantly greater incidence of UM-related mortality, shorter overall survival, and increased HR for UM-related mortality. However, the association was not retained in multivariate analysis, and the likely explanation for the link between BCVA and patient prognosis is that patients with poor vision have larger tumours. One could perhaps ask if a risk factor that does not retain its significance in multivariate analysis is truly a risk factor at all. We would argue that low BCVA can potentially be caused by several factors not directly related to tumour size and location, as mentioned in the introduction. Further, even if two risk factors correlate strongly, they might me suitable for use in different situations: E.g., if a patient presents with an intraocular tumour and low BCVA, attending optometrists and ophthalmologists can be alerted to a potentially large tumour and poor prognosis well before an ultrasonography has been performed. Lastly, independence of relevant covariates is not a requirement for risk factors in UM and other tumours, or else we would have to abandon tumour thickness as a prognostic factor, which in many cohorts is dependent of tumour diameter; and BAP-1 immunoreactivity and *BAP1* mutations, which are dependent of monosomy 3 etc.

Nevertheless, the correlation between low BCVA and larger tumours observed here is neither unexpected nor unprecedented [15]. The distribution of ciliary body involvement, tumour cell types, heavy tumour pigmentation, necrotic areas, vasculogenic mimicry, subretinal fluid, extraocular growth, tumour penetration of Bruch’s membrane, tumour infiltration of the optic nerve head, ciliary body, the iris and the anterior chamber angle was similar between patients with high and low BCVA, and none of these factors can be held responsible for the survival difference based on the findings herein [19, 26, 28, 29, 35–37, 56, 57].

We found no association between retinal detachment and poor BCVA. This highlights a limitation of this study: The data specified if detachment of any part of the retina could be observed by slit lamp biomicroscopy, but not if a retinal detachment involved the macula. Naturally, such involvement is strongly correlated with BCVA [58]. One reason for the lack of correlation in the present study may be that many of the tumour-related retinal detachments were peripheral and did not involve the central portion of the retina. Even though detection of a faint peripheral shadow may be more dependent on the extent of an intact VF and the ability to discern luminance contrast, a certain degree of retained VA may be required to be able to perceive a shadow at all. A previous study by Fili et al. found that those who presented with a shadow in the VF had better BCVA, and tumours with grater thickness and larger diameters [59].

Other limitations to this study include the limited sample used for histological correlations. It is possible that some of the correlations identified as non-significant, e.g., visual acuity and histological factors, would have been significant in a larger cohort. Not least postlaminar optic nerve invasion has previously been shown to correlate with decreased BCVA and poor survival in a larger study by Lindegaard et al. [29]. Thirdly, the risk for type II errors was further increased with Bonferroni correction, which reduced the significance level to 0.004. However, even though factors such as tumour necrosis might theoretically influence retinal function by inflammatory activity, changes in the electrolyte balance and anatomical disturbances, we are not aware that these factors have previously been shown to cause changes in BCVA independent of tumour size and location. Fourthly, we had no access to data on loss of chromosome 3 heterozygosity, *BAP1* mutations, BAP-1 expression or gene expression signatures, which are among the strongest prognostic factors in UM [60, 61]. Fifthly, our data did not include information about BCVA and the presence of disease in the contralateral eye. This may have affected patients’ perceptions of symptoms and VA, and thereby our results herein. Sixthly and last, some of our measurements of histopathological characteristics were somewhat arbitrarily chosen due to lack of precedence. Factors not investigated here may be essential to VA in UM and we make no claims to have investigated all conceivable influences.

## Conclusions

UM patients with low BCVA before primary tumour treatment have significantly worse survival, likely related to increased tumour dimensions. No significant correlations between BCVA and tumour histology including tumour cell type, degree of pigmentation, presence of necrosis, presence of subretinal fluid, extraocular growth, penetration of Bruch’s membrane, tumour infiltration of the optic nerve head or ciliary body and anterior segment involvement could be identified. Future studies should examine the prognostic significance of BCVA in relation to macula-involving retinal detachment, cytogenetic and gene expression factors.

### Summary

#### What was known before

- Visual outcomes after treatment of uveal melanoma have been investigated repeatedly. Surprisingly few studies have examined the correlation between visual acuity before primary tumour treatment with long-term patient outcomes.

#### What this study adds

- In a cohort of 1809 patients, we find that uveal melanoma patients with low visual acuity before treatment have greater incidence of uveal-melanoma-related mortality in competing risk analysis, and that they have shorter overall survival. This is likely related to the fact that patients with low visual acuity had significantly larger tumours. In the second cohort with 137 enucleated eyes, however, there were no significant relations between low visual acuity and any one of 13 examined tumour histological factors at a Bonferroni-corrected significance level.

## Supplementary information

Supplementary Table

Supplementary Figure

## Data Availability

The datasets generated during and/or analysed during the current study are available from the corresponding author on reasonable request.
