# Supplementary material for: The prognostic implication of visual acuity at the time of uveal melanoma diagnosis
Source: Eye (Lond). 2022 Nov 24;37(11):2204–11. doi: 10.1038/s41433-022-02316-8 (PMC10366190; doi:10.1038/s41433-022-02316-8)
Supplement: Supplementary file 1 — Supplementary Table [file 41433_2022_2316_MOESM1_ESM.pdf]

n	137
**Mean age at diagnosis, years (SD)**	65 (13)
**Sex, n (%)**	
Female	60 (44)
Male	77 (56)
**Tumour eye laterality, n (%)**	
Right	64 (47)
Left	58 (42)
N/a	15 (11)
**Mean tumour thickness, mm (SD)**	8.0 (3.3)
**Mean tumour diameter, mm (SD)**	14.0 (4.6)
**BCVA, mean LogMAR (SD)**	0.78 (0.68)
**BCVA, classification**	
High (LogMAR <1.00), n (%)	84 (61 %)
Low (LogMAR ≥ 1.00), n (%)	53 (39 %)
**Mean tumour distance to the optic disc, mm (SD)**	2.5 (3.2)
**Mean tumour distance to the fovea, mm (SD)**	2.7 (3.6)
**AJCC T-category, n (%)**	
1	21 (15)
2	39 (28)
3	47 (34)
4	30 (22)
**Other ocular diseases, n (%)**	
Cataract	6 (4)
Glaucoma	5 (4)
Retinal scarring	1 (<1)
Diabetic retinopathy	2 (1)

Supplementary table. Clinical characteristics of patients and tumours in the second cohort. SD, standard deviation. N/a, not available. BCVA, best corrected visual acuity of tumour eye. AJCC American Joint Committee on Cancer.
