# Supplementary material for: The prognostic implication of visual acuity at the time of uveal melanoma diagnosis
Source: Eye (Lond). 2022 Nov 24;37(11):2204–11. doi: 10.1038/s41433-022-02316-8 (PMC10366190; doi:10.1038/s41433-022-02316-8)
Supplement: Supplementary file 2 — Supplementary Figure [file 41433_2022_2316_MOESM2_ESM.pdf]

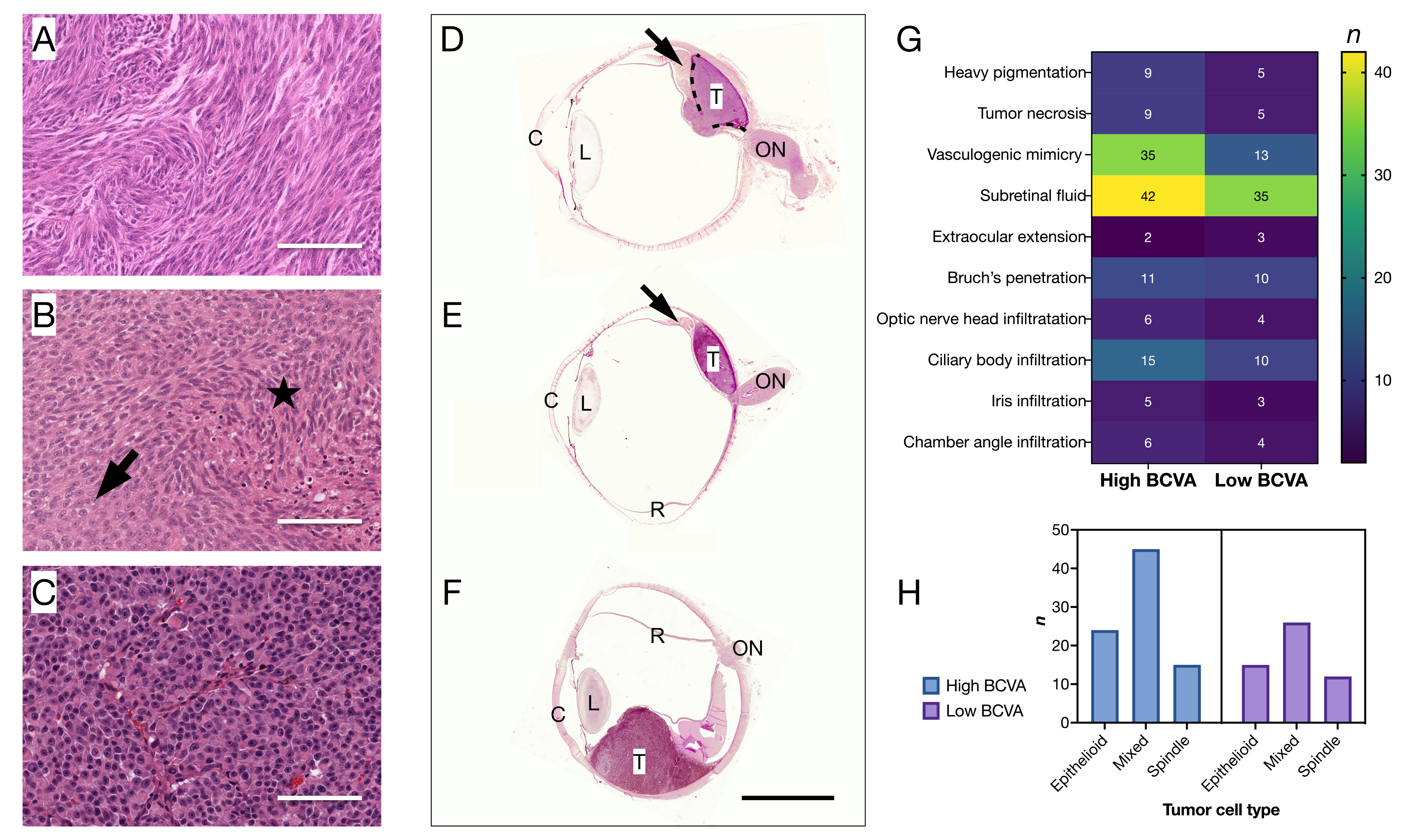

Supplementary figure. Examples of tumour histology. A) A spindle cell tumour consists of >90% spindle cells. In turn, spindle cells may be further subclassified as spindle cell type A and spindle cell type B. Spindle A cells have long, tapering cigar-like nuclei, an absent or indistinct nucleolus, and a characteristic longitudinal stripe caused by a fold in the nuclear membrane. Spindle B nuclei are oval and plumper and have less finely dispersed chromatin and a distinct nucleolus. In this study, no distinction was made between different types of spindle cells. B) A mixed tumour consists of cells with both epithelioid (arrow) and spindle-like shape (star). C) An epithelioid tumour consists of >90% epithelioid cells with round, plump nuclei, and distinct nucleoli. D) Example of a choroidal melanoma in an enucleated eye. A tumour is in close proximity to but not touching the optic disc. The mushroom or collar-button shape is a result of focal tumour penetration of Bruch's membrane. The location of Bruch's membrane is indicated (dashed lines). Fluid is present under the detached retina (arrow). Remnants of an enlarged lens can be seen. E) A small dome-shaped tumour touching the optic disc. This tumour is away from the central axis of the eye and may affect visual acuity to a lower degree than a tumour under the macula. Fluid is present near the anterior tumour base (arrow). F) A large ciliary body melanoma with significant infiltration in the choroid, heavier pigmentation, and exudative retinal detachment. G) Heat map of the number of tumours with each histopathological finding in relation to high and low BCVA. H) Bar plots, distribution of tumour cell types in relation to high and low BCVA. C, Cornea. L, Lens. ON, Optic nerve. R, Retina. T, Tumour. Scale bars A to C: 100 µm. D to F: 10 mm.
